# The healthcare costs of treating human papillomavirus-related cancers in Norway

**DOI:** 10.1186/s12885-019-5596-2

**Published:** 2019-05-07

**Authors:** Hannah Hylin, Helene Thrane, Kine Pedersen, Ivar S. Kristiansen, Emily A. Burger

**Affiliations:** 1 Department of Health Management and Health Economics, University of Oslo, PO BOX 1089, Blindern, 0137 Oslo, Norway; 2 Center for Health Decision Science, Harvard T.H. Chan School of Public Health, 718 Huntington Avenue, 2nd Floor, Boston, MA 02115 USA

**Keywords:** Human papillomavirus, Cancer, Treatment, Direct medical cost, Disease burden

## Abstract

**Background:**

Public health efforts to prevent human papillomavirus (HPV)-related cancers include HPV vaccination and cervical cancer screening. We quantified the annual healthcare cost of six HPV-related cancers in order to provide inputs in cost-effectiveness analyses and quantify the potential economic savings from prevention of HPV-related cancers in Norway.

**Methods:**

Using individual patient-level data from three unlinked population-based registries, we estimated the mean healthcare costs 1) annually across all phases of disease, 2) during the first 3 years of care following diagnosis, and 3) for the last 12 months of life for patients diagnosed with an HPV-related cancer. We included episodes of care related to primary care physicians, specialist care (private specialists and hospital-based care and prescriptions), and prescription drugs redeemed at pharmacies outside hospitals between 2012 and 2014. We valued costs (2014 €1.00 = NOK 8.357) based on diagnosis-related groups (DRG), patient copayments, reimbursement fees and pharmacy retail prices.

**Results:**

In 2014, the total healthcare cost of HPV-related cancers amounted to €39.8 million, of which specialist care accounted for more than 99% of the total cost. The annual maximum economic burden potentially averted due to HPV vaccination will be lower for vulvar, penile and vaginal cancer (i.e., €984,620, €762,964 and €374,857, respectively) than for cervical, anal and oropharyngeal cancers (i.e., €17.2 million, €6.7 million and €4.6 million, respectively). Over the first three years of treatment following cancer diagnosis, patients diagnosed with oropharyngeal cancer incurred the highest total cost per patient (i.e. €49,774), while penile cancer had the lowest total cost per patient (i.e. €18,350). In general, costs were highest the first year following diagnosis and then declined; however, costs increased rapidly again towards end of life for patients who did not survive.

**Conclusion:**

HPV-related cancers constitute a considerable economic burden to the Norwegian healthcare system. As the proportion of HPV-vaccinated individuals increase and secondary prevention approaches advance, this study highlights the potential economic burden avoided by preventing these cancers.

**Electronic supplementary material:**

The online version of this article (10.1186/s12885-019-5596-2) contains supplementary material, which is available to authorized users.

## Background

Human papillomavirus (HPV) is a known carcinogen for cancer of the cervix, vagina, vulva, penis, anal canal and oropharynx, and accounts for approximately 5% of all cancers globally [1]. In Norway, between 2010 and 2014, approximately 2% of all cancer patients (i.e., over 600 patients) were diagnosed with an HPV-related cancer each year [2], the majority of which were cervical or oropharyngeal cancers (i.e., ~ 300 and ~ 150 cases, respectively).

Prevention of HPV-related cancers in Norway involves primary (i.e., HPV vaccination) and secondary (i.e., cervical cancer screening) approaches. Nationwide, organized, cytology-based cervical cancer screening has been ongoing since 1995. Starting in 2018, primary HPV testing will replace cytology for women aged 34 years and older, which is expected to improve the effectiveness and efficiency of the program [3, 4]. Since 2009, school-based HPV vaccination has been offered to 12-year-old girls, and in 2016, primary prevention efforts were expanded to include a temporary ‘catch-up’ HPV vaccination campaign for women aged 26 years and younger (who did not receive the HPV vaccine in adolescence). Starting the fall of 2018, the routine HPV vaccination program will be expanded to include adolescent boys aged 12 years [5].

Although previous studies have evaluated the resource use associated with HPV-related diseases in Norway [6–8], two of these studies [6, 7] relied on national guidelines, expert opinion and aggregated registry data to quantify and value the societal costs of treating HPV-related diseases. Oslo Economics [8] used individual registry data to quantify the total cost of the most common cancers in Norway, including cervical cancer, but did not quantify treatment costs for the five remaining HPV-related cancers. Among similar studies in neighboring Scandinavian countries, one study evaluated the incidence and hospital-related costs of four of the six HPV-related cancers in Denmark [9], while a recent Swedish study quantified the economic burden of HPV-related cancers one year prior to the implementation of HPV vaccination [10]. To our knowledge, no studies have used individual-level data from national health registries to quantify the economic burden of all HPV-related cancers in Norway or provide estimates of the costs during the different phases of care. In order to gain knowledge of the type of care patients with HPV-related cancers receive, as well as to inform future policy analyses, we aimed to perform a descriptive study to assess the overall healthcare costs of HPV-related cancers in Norway, and the distribution of such costs within the healthcare system. This study complements the above-mentioned Danish [9] and Swedish [10] studies, and will therefore contribute to a complete Scandinavian status update for this patient group.

## Methods

We estimated the healthcare costs of HPV-related cancers in Norway by identifying the resource use directly linked to formal patient care [11]. From the Norwegian context, formal patient care costs primarily include the costs related to: inpatient and outpatient care in somatic hospitals, outpatient care with private specialist, primary care physicians, and costs related to prescription drug use outside the hospital setting. To identify and quantify resource use, we determined the number of patients with an HPV-related diagnosis from three unlinked Norwegian national patient registries. To value the resource use, we applied a mean costing approach using national tariffs, assuming that mean costs reflect the marginal costs in the long term [11]. We valued all costs in 2014 Norwegian Kroner (NOK), and converted to 2014 Euros (mean annual exchange rate €1 = NOK 8.357) [12].

### Study setting and data

We selected the study population from three unlinked Norwegian national patient registries. 1) The Norwegian Patient Registry (NPR), which includes patients who received inpatient and outpatient care in somatic hospitals between 2008 and 2014, 2) The Norwegian Control and Distribution of Health Reimbursement Database (KUHR), which includes patients who received care from private specialists and/or primary care physicians (PCPs) between 2009 and 2015, and 3) the Norwegian Prescription Registry (NorPD), which includes patients who redeemed prescription drugs from pharmacies (outside the hospital) between 2009 and 2014. From each of the three registries, we identified the number of patients with an HPV-related diagnosis using the registry diagnostic codes, i.e., the International Classification of Disease (ICD10) C01, C09, C10, C21, C51, C52, C53 and C60 and International Classification of Primary Care (ICPC2) D75, D77, X75 and U77 (Additional file 1: Table S1).

As each patient may be registered with more than one main diagnosis across episodes of care in our unlinked registry data sets, we applied multiple approaches to assign a main diagnosis that ultimately informed the final main diagnosis (see Part II in Additional file 1 for more details and Additional file 1: Table S2). This multi-step diagnosis assignment approach was similar to the approach applied in a previous costing analysis [8]. Following main diagnosis assignment, we included only those patients with a specific HPV-related diagnostic code as the main diagnosis (Additional file 1: Table S1, Figure S1).

Following identification of patients with an HPV-related cancer as a main diagnosis, we formed distinct sub-samples for three different analytic purposes: 1) the ‘general cost analysis’ that estimated the mean aggregated annual costs across all phases of care 2) the ‘incidence-based cost analysis’ that estimated costs in the first, second and third years following diagnosis, and 3) the ‘end-of-life cost analysis’ that estimated costs with proximity to death. For the ‘general costing analysis,’ we included all patients with episodes of care occurring between January 1^st^, 2012 and December 31^st^, 2014. Although registry data for years 2009–2011 were available for all data sets, we excluded those data due to substantial changes in the registration of outpatient care and radiation therapy in the Diagnostic Related Groups (DRG) system. For the ‘incidence-based cost analysis’, we included patients with their first episode of care after December 31^st^, 2011 (i.e., no observations between 2008 and 2011 in order to restrict analyses to include probable newly diagnosed cancers). Finally, for our ‘end-of-life cost analysis’, we included observations for patients’ last 12 months of life, among patients who had received treatment for more than one year. While the ‘general costing analysis’ included observations from all three registries, the ‘incidence-based cost analysis’ and ‘end-of-life cost analysis’ included observations from inpatient and outpatient hospital episodes (i.e., NPR), as this was the only registry that provided information related to date of diagnosis and days prior to death.

We applied an approach similar to activity-based costing (ABC) for measuring costs. Both ABC and time-driven ABC are more commonly applied in cancer cost analyses as they provide more accurate cost information about complexed systems where the main resource is a skilled work force [13].

For somatic hospital care, we estimated costs by multiplying the DRG-weights by the value of one 2014 DRG (i.e., €4879) for each registered episode of care. For all outpatient consultations, we added a cost of €38 to reflect the 2014 patient copayment rate [14]. To account for costs not covered by the DRG weight (i.e., capital cost, ambulance cost, pensions, other laboratory and radiology services), we added 22.17% of the episode cost to each episode of care, representing a previously estimated proportion of the cost of these services [8] (Additional file 1: Table S3).

For episodes of care related to primary care physicians, private specialists and prescription drugs redeemed at pharmacies, we estimated costs based on patient copayments, reimbursement fees and pharmacy retail prices (excluding value added tax (VAT) [8, 11]) (Additional file 1: Table S3 and S4). For each primary care physician visit, we supplemented each episode of care with a cost of €16 to reflect the mean per capita fee per episode of care. For private specialists, we supplemented each episode of care with a cost of €49 to reflect the mean specialist practice allowance per episode of care.

### Analysis

For the ‘general cost analysis’, we estimated the total annual healthcare cost per HPV-related cancer as well as disaggregated by service level: specialist care (i.e., inpatient hospital, outpatient hospital and private specialists), PCPs, and by prescription drugs redeemed in pharmacies between 2012 and 2014. For this analysis, we also apportioned the HPV-related cancers that are likely to be directly linked to an HPV infection and potentially preventable by the Norwegian HPV vaccination program, i.e., “HPV-attributable cancers”. To calculate the total HPV-attributable costs in specialist care in 2014, we assumed that the proportion of cancer cases attributable to HPV were the same as those presented by Hansen and colleagues [15] (Table 2). This assumption was not transferred to the primary physician care setting or the prescription drugs redeemed in pharmacies because there is no guarantee that all cancer patients are in contact with primary physician care or pharmacy during their treatment period. As uncertainty exists around the attributable proportion of HPV-positive cancers, in sensitivity analysis we used a large, multi-site study from the United States [16] to assign HPV-positive cancers. We further disaggregated per patient costs within somatic care (i.e., inpatient and outpatient care) by accounting for the cost per patient in 2014.

For the ‘incidence-based cost analysis’, we estimated the cost of care per patient in somatic hospitals for the first, second, and third years following cancer diagnosis between 2012 and 2014. For this analysis, we assumed that patients without any cancer diagnosis during the preceding four years received their initial HPV-related cancer diagnosis between 2012 and 2014.

Finally, for the sub-sample of patients who died between 2012 and 2014, we identified the cost of care during the patients’ last 12 months of life. Assuming treatment following an initial cancer diagnosis is more costly than subsequent treatment, we excluded all patients who had less than 12 months of observation time following their first episode of care in the registry.

Data were analysed using Stata version 14.1 and Microsoft Excel 2016.

### Ethics and approval

NPR, KUHR and NorPD were analyzed with anonymized patient IDs, and were not linked. The study was approved by the Norwegian Center for Research Data (project reference 52580).

## Results

### Study population

**Fig. 1:**
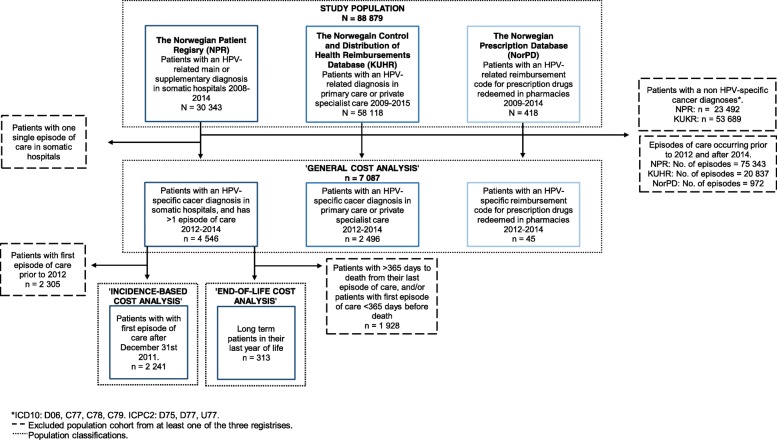
Flow-chart of inclusion and exclusion of patients with HPV-related cancer in three population-based Norwegian registries. Overview of patients included in each of the three population-based patient registries: The Norwegian Patient Registry (NPR), The Norwegian Control and Distribution of Health Reimbursement Database (KUHR) and The Norwegian Prescription Database (NorPD). The exclusion criteria applied varied with the type of registry. We constructed three sub-samples: 1) the ‘General Cost Analysis’, which included patients registered with a HPV-specific main diagnosis in any registry between between 2012 and 2014, 2) for the ‘Incidence-based Cost Analysis’, which included patients in NPR with an HPV-related main diagnosis who had their first episode of care after December 31^st^, 2011, and 3) the ‘End-of-life Cost Analysis’, which included patients in NPR with an HPV-related main diagnosis who had started treatment at least 12 months prior to dying in between 2012 and 2014

Using NPR (i.e., inpatient and outpatient hospital episodes of care), we identified 30,343 unique patients with an HPV-related main or supplementary diagnosis in somatic hospitals between 2008 and 2014, of which 4546 patients were eligible for study inclusion (Fig. 1). Primary reasons for excluding 27,013 patients included: 1) patients had a non-specific HPV-related main diagnosis (i.e., ICD10 codes C77, C78, C79 and Z51), or 2) patients were registered with only one episode of care during the given period. During our analytic time horizon (2012–2014), we identified a total of 2055 patients with cervical cancer, 57 patients with vaginal cancer, 49 patients with vulvar cancer, 312 patients with penile cancer, 629 patients with anal cancer and 1024 patients with oropharyngeal cancer. Among the 4546 identified eligible patients in somatic hospitals, 2241 patients were newly diagnosed (i.e., with no episodes of care prior to 2012), comprising the sub-sample for the ‘incidence-based cost analysis’. We also identified 313 patients who died between 2012 and 2014, comprising the sub-sample for the ‘end-of-life cost analysis’.

From KUHR (private specialists and/or PCP care), we identified 58,118 unique patients with an HPV-related main or supplementary diagnosis between 2009 and 2015, of which 2496 patients were eligible for study inclusion during our analytic time horizon (2012–2014). Finally, from NorPD (prescription drug use) we identified 418 unique patients with an HPV-related reimbursement code for prescription drugs redeemed in pharmacies between 2009 and 2014, of which 45 patients were diagnosed (all with anal cancer) between 2012 and 2014 and were eligible for study inclusion. In total, we identified 7087 individual patients from the three registries (which were not necessarily unique patients, as the registries were not linked).

### Total annual healthcare costs

The total annual healthcare costs for the six HPV-related cancers increased from €35.5 million in 2012 to €39.8 million in 2014, reflecting a 12% increase in costs measured in constant prices over the 3-year period (Table 1).

**Table 1:** Total healthcare cost (€) of HPV-related cancers according to cancer diagnosis, year and type of healthcare, 2012–2014, €1.00 = NOK 8.357

		Type of healthcare						
Cancer diagnosis	Year	Specialist care^a^		Primary physician care		Prescription drugs		Total healthcare cost
		Cost (€)	% of total healthcare cost	Cost (€)	% of total healthcare cost	Cost (€)	% of total healthcare cost	
Cervical cancer	2012	13,521,776	*98.49*	207,603	*1.51*	–	–	13,729,379
	2013	15,542,183	*98.57*	225,436	*1.43*	–	–	15,767,619
	2014	17,213,190	*98.66*	234,055	*1.34*	–	–	17,447,245
Vaginal cancer	2012	455,472	*100.00*	–	–	–	–	455,472
	2013	430,787	*100.00*	–	–	–	–	430,787
	2014	462,786	*100.00*	–	–	–	–	462,786
Vulvar cancer	2012	3,454,485	*100.00*	–	–	–	–	3,454,485
	2013	3,175,389	*100.00*	44	*0.00*	–	–	3,175,433
	2014	3,395,240	*100.00*	–	–	–	–	3,395,240
Penile cancer	2012	1,309,498	*100.00*	–	–	–	–	1,309,498
	2013	1,397,461	*100.00*	–	–	–	–	1,397,461
	2014	1,623,327	*100.00*	–	–	–	–	1,623,327
Anal cancer	2012	4,807,059	*99.42*	62	*0.00*	27,785	*0.57*	4,834,906
	2013	4,578,332	*99.19*	–	–	37,328	*0.81*	4,615,660
	2014	5,108,420	*99.72*	23	*0.00*	14,597	*0.28*	5,123,040
Oropharyngeal cancer	2012	11,748,410	*100.00*	91	*0.00*	–	–	11,748,501
	2013	11,622,064	*100.00*	77	*0.00*	–	–	11,622,141
	2014	11,736,770	*100.00*	–	–	–	–	11,736,770
All HPV-related cancers	2012	35,296,699	*99.34*	207,756	*0.59*	27,785	*0.08*	35,532,240
	2013	36,746,215	*99.29*	225,557	*0.61*	37,328	*0.10*	37,009,100
	2014	39,539,733	*99.38*	234,077	*0.59*	14,597	*0.04*	39,788,406
*Mean across 2012–2014*		*37,194,216*		*222,463*		*26,570*		*37,443,249*
*Percentage increase, 2012–1014*		*12.02*		*12.67*		*−47.46*		*11.98*

^a^Specialist care includes the cost of somatic hospitals and private specialists

Healthcare provided by somatic hospitals (both inpatient and outpatient care) accounted for more than 99% of the total healthcare costs of HPV-related cancers during the study period. Due in part to the unique burden of each HPV-related cancer, there were substantial differences in total cost between the cancer types. For example, the total annual healthcare cost was highest for cervical cancer, (ranging from €13.7 million in 2012 to €17.4 million in 2014), and lowest for vaginal cancer (€455,000 in 2012 and €463,000 in 2014). HPV-attributed cancers (those attributed directly to an HPV infection) accounted for approximately 77% of the total HPV-related health care costs in specialist care (€30.6 million in 2014) (Table 2; left panel). When we explored the proportions of HPV-attributable cancers using the study by Saraiya and Colleagues [16], we found that although there were some deviations between cancers types, the total economic burden of HPV-attributable cancers remained similar, i.e., €31,897,358 (Table 2; right panel).

**Table 2:** Total HPV-attributable cancer cost (€) in specialist care according to cancer diagnosis, 2014, € 1.00 = NOK 8.357

Cancer diagnosis	HPV-related total costs (€)	Base Case Analysis (Hansen et al. 2015) [15]		Sensitivity Analysis (Saraiya et al. 2015) [16]	
		Proportion attributable to HPV (%)	HPV-attributable total costs (€)	Proportion attributable to HPV (%)	HPV-attributable costs (€)
Cervical cancer	17,213,190	100	17,213,190	90.6	15,595,150
Vaginal cancer	462,786	81	374,857	75	347,090
Vulvar cancer	3,395,240	29	984,620	68.8	2,335,925
Penile cancer	1,623,327	47	762,964	63.3	1,027,566
Anal cancer	5,108,420	90	4,597,578	90.6^a^	4,628,229
Oropharyngeal cancer	11,736,770	57	6,689,959	67.85^a^	7,963,398
Total burden of HPV	39,539,733		30,623,167		31,897,358
*Proportion of HPV-related total cost (%)*			*77.45*		*80.67*

^a^Average HPV-attributable proportion. Originally reported as gender specific rates in Saraya et al. 88.7% (male) and 92.6% (female) for anal cancer and 63.3% (female) and 72.4% (male) for oropharyngeal cancer

The economic burden potentially averted due to HPV vaccination will be lower for vulvar, penile and vaginal cancer (i.e. €984,620, €762,964 and €374,857 per year, respectively) than for cervical, oropharyngeal and anal cancers (i.e. €17.2 million, €6.7 million and €4.6 million, respectively).

### Cost per patient in somatic hospitals

The annual cost per patient in somatic hospitals differed substantially between the HPV-related cancers (Table 3).

**Table 3:** Annual mean cost per patient (€) according to type of care and cancer type, 2014, €1.00 = NOK 8.357

Cancer diagnosis	Cost (€) per patient			
Type of care	No. of patients^a^	Mean	Std.	Median
Cervical cancer				
Somatic hospital care	1213	14,130	21,857	1369
*Inpatient*	*464*	*21,278*	*18,280*	*17,583*
*Outpatient*	*1163*	*6248*	*12,828*	*981*
Vaginal cancer				
Somatic hospital care	33	14,005	15,488	7150
*Inpatient*	*18*	*17,701*	*12,001*	*16,822*
*Outpatient*	*32*	*4486*	*7447*	*994*
Vulvar cancer				
Somatic hospital care	233	14,523	19,724	4606
*Inpatient*	*125*	*23,893*	*19,273*	*22,617*
*Outpatient*	*215*	*1848*	*3938*	*750*
Penile cancer				
Somatic hospital care	190	8537	16,522	1511
*Inpatient*	*72*	*19,271*	*21,170*	*8629*
*Outpatient*	*178*	*1318*	*1949*	*713*
Anal cancer				
Somatic hospital care	401	12,736	20,621	2027
*Inpatient*	*154*	*23,599*	*23,298*	*16,461*
*Outpatient*	*388*	*3796*	*5051*	*1426*
Oropharyngeal cancer				
Somatic hospital care	714	16,432	26,550	892
*Inpatient*	*247*	*33,593*	*27,355*	*26,867*
*Outpatient*	*698*	*4921*	*8347*	*717*

^a^The number of inpatients and outpatients do not represent unique patients because patients can receive care multiple times, and may therefore be coded as both inpatient and outpatient dependent on which type of care they receive

Oropharyngeal treatment required the highest cost per patient (i.e., €16,432), whereas penile cancer treatments required the lowest (i.e., €8537). Inpatient care was more resource consuming than outpatient care. For example, in 2014 for cervical cancer patients, the cost for inpatient care was €21,278 per patient, whereas outpatient care for these patients was €6248 per patient.

### Cost per patient during the first three years of diagnosis

For all HPV-related cancers diagnosed between 2012 and 2014 in somatic hospitals, the mean cost per patient per year was higher the first year of diagnosis than the second and third years, accounting for approximately 90% of the total cost over the 3-year period (Table 4).

**Table 4:** Cost per patient (€) the first, second and third year of diagnosis according to cancer type, 2012–2014, €1.00 = NOK 8.357^a^

	Cost (€) per patient			
Cancer diagnosis by year of treatment^b^	No. of patients	Mean	Std.	Median
Cervical cancer				
1st	954	32,220	27,989	26,324
Proportion of total cost first 3 years (%)		(90.71)		
2nd	290	8989	20,572	892
3rd	99	5436	13,660	375
Total cost first 3 years	954	35,518	33,556	27,358
Vaginal cancer				
1st	28	30,210	15,234	30,971
Proportion of total cost first 3 years (%)		(97.68)		
2nd	7	2712	5747	500
3rd	2	563	265	563
Total cost first 3 years	28	30,928	15,352	33,782
Vulvar cancer				
1st	224	23,798	22,066	20,024
Proportion of total cost first 3 years (%)		(90.61)		
2nd	53	8550	18,035	731
3rd	13	7655	12,501	487
Total cost first 3 years	224	26,265	26,342	22,523
Penile cancer				
1st	141	16,085	21,828	8222
Proportion of total cost first 3 years (%)		(87.66)		
2nd	38	8311	21,397	713
3rd	9	396	119	356
Total cost first 3 years	141	18,350	26,245	8466
Anal cancer				
1st	330	26,876	25,934	21,692
Proportion of total cost first 3 years (%)		(88.83)		
2nd	111	8221	18,460	1426
3rd	43	4720	11,079	951
Total cost first 3 years	330	30,256	31,223	23,415
Oropharyngeal cancer				
1st	564	48,055	37,376	41,362
Proportion of total cost first 3 years (%)		(96.55)		
2nd	215	3965	11,619	536
3rd	77	1515	8244	212
Total cost first 3 years	564	49,774	39,737	41,651

^a^The patient population represent the incidence population

^b^Year of treatment reflects treatment year

Over the first three years of treatment following cancer diagnosis, patients diagnosed with oropharyngeal cancer incurred the highest total cost per patient (i.e., €49,774), while penile cancer had the lowest total cost per patient (i.e., €18,350).

### Cost per patient during last year of life

**Fig. 2:**
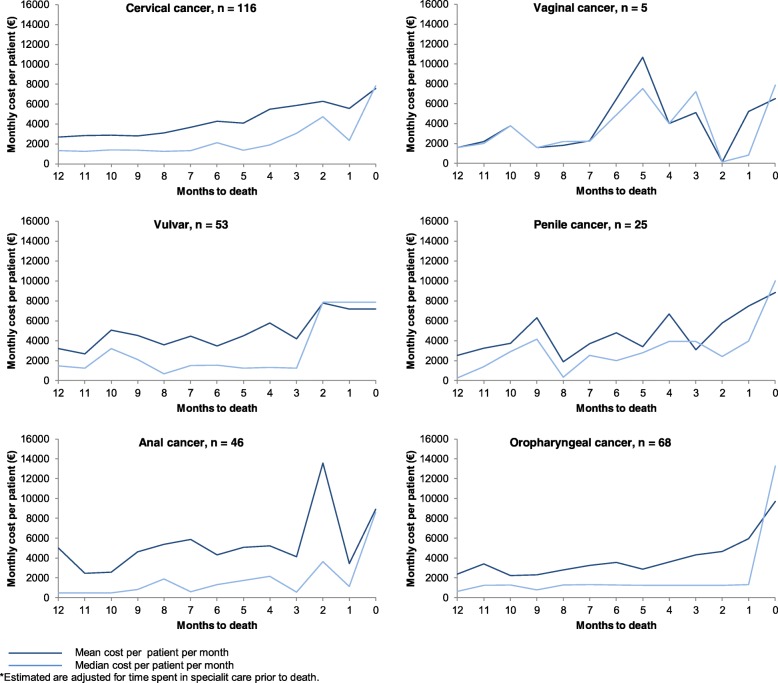
Cost per patient with proximity to death. Mean (dark blue) and median (light blue) monthly cost (€) per patient during the last year of life, according to cancer diagnosis and treatment month in proximity to death. Graphs are based on the sub-sample for the ‘End-of-life Cost Analysis’ analytic cohort (Fig. 1)

In general, the mean monthly cost of care for patients in somatic hospitals between 2012 and 2014 increased with proximity to death (Fig. 2). The increase was especially evident in the last three months of life for patients with cervical cancer, vulvar cancer and oropharyngeal cancer. For example, the mean monthly cost of care for terminally ill patients with cervical cancer, increased steadily from €3000 per patient 12 months prior to death, to €8000 per patient the month directly preceding death. Terminally ill patients with vulvar or oropharyngeal cancers had similar increases, (i.e., from €4000 to €8000 and €4000 to €9000 per patient, respectively). In contrast, the cost per patient with vaginal cancer, penile cancer and anal cancer did not reflect the same trend as the costs varied largely from one month to the next during the entire 12-month period, in part due to small sample sizes (e.g., vaginal cancer).

## Discussion

In Norway between 2012 and 2014, the total healthcare cost of treating HPV-related cancers was approximately €40 million per year, of which €30.6 million may be directly preventable through HPV vaccination. In 2014, HPV-related cancers accounted for 2.29% of the total cancer-related costs in Norway and 0.11% of the total Norwegian healthcare costs [8]. We estimated that specialist care accounts for approximately 99% of the total healthcare cost, while primary physician care and outpatient prescriptions together accounted for less than 1%. As expected, the healthcare cost of hospital care was higher for inpatient care compared with outpatient care. We also found that the treatment costs for the first year following diagnosis was higher compared to the second and third years, but the cost of care increased again with proximity to death.

To our knowledge, this is the first Norwegian study to evaluate the healthcare cost of HPV-related cancers in Norway using comprehensive individual-level population-based data. Compared to the previous Norwegian study by Burger and colleagues [7] our analysis estimated a higher cost per patient for the first three years of diagnosis. Differences in estimates may be in part explained by the application of registry-based data that reflects actual resource use, while Burger and colleagues [7] estimated the expected costs associated with national treatment guidelines. Our results were to a certain degree consistent with other Scandinavian studies in the rank-order of the most- to least-costly HPV-related cancer, but also deviated in the magnitude of the costs [9, 10]. For example, Olsen and colleagues [9] estimated the cost per patient of vulvar cancer the first, second and third years following diagnosis to be €13,688, €4481 and €4760 (2008€), respectively, which are lower than our estimates. However, their estimated cost per patient with anal cancer were similar to ours (i.e., €26,104, €8783 and €6375 the first, second and third years, respectively). Östensson et al. [10] presented mean cost per health care episode for all inpatient and outpatient care, at Swedish hospitals during 2006. In Sweden (2006), the average cost for cervical cancer were €6063 per patient episode for inpatient care and €346 per patient episode in outpatient care. While the Swedish study by Östensson et al. presented health care episode costs during a 1-year period, our study presented health care costs for specific patients during a 3-year period, which contributes to the somewhat higher costs presented in our analysis. Further, in our analysis we applied “DRG” costs while the Swedish study used a “cost-per-patient” approach. The use of different methods could also explain why the costs are higher in this analysis in comparison with the Swedish study.

Within somatic hospitals, inpatient care resulted in higher treatment costs than outpatient care, which is expected as it generally involves more invasive treatment such as surgical treatment and more comprehensive diagnostics. The findings by Östensson and colleagues [10] are in line with this finding. We also found that the cost per patient decreases from the first, to the second and third years of diagnosis. Findings by Olsen and colleagues [9] and other studies investigating other cancer diagnoses [17–20] also report this trend. Similar to other studies, we found that the monthly cost per patient increased with proximity to death for patients with cervical, vulvar and oropharyngeal cancers. These results are in accordance with another Norwegian registry-based analysis investigating other cancers [8]. Nevertheless, use of chemotherapy towards the end of life should be subject to further research as the treatment can be costly, in terms of both monetary cost and side effects [21].

In general, differences in estimates between existing cost studies may be due to differences in the organization of the healthcare system, medical practices and unit costs. These differences underscore the importance of country-specific analyses, even between seemingly similar Scandinavian countries. Our results, therefore, may not be applicable to other countries; however, certain trends may be generalizable.

Our study has several limitations. First, the method for assigning one diagnosis to each patient introduced uncertainty to our analysis because some patients had multiple cancer diagnoses. However, different approaches to assign a cancer diagnosis to each patient resulted in similar assignments (Additional file 1: Table S2). We further minimized the uncertainty of whether the costs derived from an HPV-related cancer by only including patients with a specific HPV-related diagnosis. Nevertheless, future analyses may be able to reduce diagnosis uncertainty by linking the patient-level health care data to information on cancer diagnosis from the Cancer Registry of Norway. This approach would enable an analysis that follows individual patients through entire primary and specialist care pathways, as well their use of pharmaceuticals. Second, we were unable to adjust the costs for the patients’ cancer stage at diagnosis. Third, there is uncertainty with respect to DRG weights and whether they capture all hospital costs. In order to account for capital costs, pensions and some laboratory and radiology services not included in the DRG costs, we supplemented each episode of care with a percentage increase (i.e., 22.17%) based on estimations from a previous Norwegian report on cancer costs [8]. Lastly, the direct medical costs of HPV-related cancer care only represent a proportion of the total societal burden of HPV-related cancers, particularly considering production loss due to sick leave and premature death as estimated by Pedersen and colleagues [6]. Future registry-based analyses should evaluate the broader societal perspective and include costs outside the healthcare sector. Accounting for these costs would provide a comprehensive measure of the total societal burden potentially averted due to existing primary and secondary prevention efforts in Norway.

### Policy implications

Quantifying the economic burden of HPV-related cancers, including the type and intensity of treatment, is essential to understand the burden of disease and ensure efficient resource allocation. Although cost of illness studies do not inform priority setting, this study can provide essential information for future cost-effectiveness analyses of HPV-prevention. The cost of these cancers can represent future potential cost savings of HPV prevention strategies, such as cervical cancer screening and vaccination programmes. Ultimately, as the proportion of HPV-vaccinated individuals increase and secondary prevention approaches advance, the health economic burden of treating HPV-related cancers is expected to decline.

## Conclusion

HPV-related cancers constitute a considerable economic burden to the Norwegian healthcare system with an estimated annual total mean healthcare cost of €37.4 million per year. This study highlights the potential economic burden avoided by preventing these cancers.

## Additional file

Additional file 1: Supplementary Appendix (DOCX 110 kb)

## Abbreviations

DRG: Diagnostic related groups
HPV: Human papillomavirus
KUHR: The Norwegian Control and Distribution of Health Reimbursement Database
NorPD: Norwegian Prescription Database
NPR: Norwegian Patient Registry
