# Supplementary material for: The healthcare costs of treating human papillomavirus-related cancers in Norway
Source: BMC Cancer. 2019 May 7;19:426. doi: 10.1186/s12885-019-5596-2 (PMC6505196; doi:10.1186/s12885-019-5596-2)
Supplement: Supplementary file 1 — Supplementary Appendix (DOCX 110 kb) [file 12885_2019_5596_MOESM1_ESM.docx]

**SUPPLEMENTARY APPENDIX**

Accompanying the manuscript:

**The healthcare cost of human papillomavirus-related cancers in Norway**

Hannah Hylin, MPhil*

Helene Thrane, MPhil*

Kine Pedersen, PhD

Ivar S. Kristiansen, MD, MPH, PhD

Emily A. Burger, PhD

*Denotes equal contribution

Part I: Diagnostic codes

Part II: Diagnosis assignment

Part III: Cost components

Part IV: References

**Part I: Diagnostic codes**

In specialist care, the use of International statistical classification of diseases and other health problems (ICD-10) codes, have become the international standard for describing health states [1]. ICD-10 codes are the diagnostic codes reported from the hospital to NPR (Appendix Table 1).

The International Classification of Primary Care (ICPC-2) is the standard clinical code for primary care in Norway [2]. ICPC-2 is sufficient in addressing the main elements of general medical practice, such as in KUHR, but the codes are too wide and inexact to be applied in specialist care [3]. We excluded four ICPC-2 codes from our analysis because the described location of the cancer was too ambiguous (Appendix Table 1). KUHR operates with both ICD-10 codes and ICPC-2 codes.

Reimbursement codes for prescription drugs are denoted by either ICD-10 codes, ICPC-2 codes or numerical codes. Numerical codes were not applied in our analysis. The use of ICD-10 codes are usually, but not always from the specialist care, while prescriptions with ICPC-2 reimbursement codes are stem from the primary care (Appendix Table 1).

**Additional file 1: Table S1** Relevant available diagnostic codes and reimbursement codes, according to registry, clinical code scheme, definition, associated cancer and reimbursement eligibility.

| **Registry** | **Clinical code scheme** | **Code** | **Definitionᵃ** | **Associated cancer** |  |
| --- | --- | --- | --- | --- | --- |
| NPR | ICD-10 | C01 | Malignant neoplasm of base of tongue | Oropharyngeal cancer |  |
|  |  | C09 | Malignant neoplasm of tonsil | Oropharyngeal cancer |  |
|  |  | C10 | Malignant neoplasm of oropharynx | Oropharyngeal cancer |  |
|  |  | C21 | Malignant neoplasm of anus and anal canal | Anal cancer |  |
|  |  | C51 | Malignant neoplasm of vulva | Vulvar cancer |  |
|  |  | C52 | Malignant neoplasm of vagina | Vaginal cancer |  |
|  |  | C53 | Malignant neoplasm of cervix uteri | Cervical cancer |  |
|  |  | C60 | Malignant neoplasm of penis | Penile cancer |  |
|  |  | C77 | Secondary and unspecified malignant neoplasm of lymph nodes | Not cancer specific |  |
|  |  | C78 | Secondary malignant neoplasm of respiratory and digestive organs | Not cancer specific |  |
|  |  | C79 | Secondary malignant neoplasm of other and unspecified sites | Not cancer specific |  |
|  |  | Z51 | Encounter for other aftercare and medical care | Not cancer specific |  |
| KUHR | ICD-10 | C01 | Malignant neoplasm of base of tongue | Oropharyngeal cancer |  |
|  |  | C09 | Malignant neoplasm of tonsil | Oropharyngeal cancer |  |
|  |  | C10 | Malignant neoplasm of oropharynx | Oropharyngeal cancer |  |
|  |  | C21 | Malignant neoplasm of anus and anal canal | Anal cancer |  |
|  |  | C51 | Malignant neoplasm of vulva | Vulvar cancer |  |
|  |  | C52 | Malignant neoplasm of vagina | Vaginal cancer |  |
|  |  | C53 | Malignant neoplasm of cervix uteri | Cervical cancer |  |
|  |  | C60 | Malignant neoplasm of penis | Penile cancer |  |
|  | ICPC-2 | D75* | Malignant neoplasm of colon/rectum | Anal cancer |  |
|  |  | D77* | Malignant neoplasm of digestive system | Oropharyngeal cancer |  |
|  |  | X75 | Malignant neoplasm of cervix uteri | Cervical cancer |  |
|  |  | X81* | Genital neoplasm unspecified | Vaginal and vulvar cancer |  |
|  |  | U77* | Malignant neoplasm urinary other | Penile cancer |  |
|  |  |  |  |  | **Reimbursement codeᵇ** |
| NorPD | ICD-10 | C21 | Malignant neoplasm of anus and anal canal | Anal cancer | Yes |
|  | ICPC-2 | D75* | Malignant neoplasm of colon/rectum | Anal cancer | Yes |
|  |  | D77* | Malignant neoplasm of digestive system | Oropharyngeal cancer | Yes |
| *Codes omitted from cost analysis due to ambiguous location of cancer. ᵃfinnkode.no ᵇlegemiddelsok.no | | | |  |  |
| Abbreviations: KUHR, The Norwegian Control and Distribution of Health Reimbursement Database; NorPD, The Norwegian Prescription Registry; NPR, The Norwegian Patient Registry. | | | | | |

**Part II: Diagnosis assignment**

With the aim of assigning one main diagnosis per patient, we applied five different methods to yield intermediary results, that would inform a final main diagnosis. Oslo Economics [4] in cooperation with a group of experts defined four approaches on how to assign one cancer diagnosis to each patient, based on an NPR data set. The different approaches valued the frequency of diagnosis across all episodes of care, and episodes of care occurring the last calendar year. We added a fifth approach as a supplement to the four identified by Oslo Economics, where we accounted for diagnostic codes earlier in the treatment course (Appendix Table 2).

The main mechanism behind the assignment of one main diagnosis per patient was frequency of diagnosis. Therefore, we experienced ties between two or more diagnosis for a small share or patients. In the event of ties, we assigned the last occurring diagnosis in the respective calendar year or across all years (Appendix Table 2).

All five diagnosis assignments were only applied to the NPR dataset. We were unable to apply Diagnosis assignment C and D to KUHR as the data set does not provide supplementary diagnosis. Furthermore, diagnosis assignment to patients in NorPD was only based on Diagnosis assignment A, because there was such a wide spread in reimbursement codes in the data set.

Once each patient was assigned one main diagnosis, we wanted to minimize the uncertainty of whether the patients’ healthcare costs derived from an HPV-related cancer. Therefore, we divided the patients into three groups, where the different groups reflected the certainty of the costs deriving from an HPV-related cancer:

*Patient group I:* Patients with a specific HPV-related diagnostic code as main diagnosis.

Patient group I represented the patients whose healthcare costs most likely derived from an HPV-related cancer, and included patients from NPR, KUHR and NorPD. Only patients in Patient group I were included for cost analysis.

*Patient group II:* Patients with an unspecific HPV-related diagnostic code as main diagnosis.

Patient group II included patients from both KUHR and NorPD with an unspecific ICPC-2 diagnosis (specified in Appendix Table 1). Patients in Patient group II were disregarder for further cost analysis.

*Patient group III:* Patients with a non-HPV-related diagnostic code as main diagnosis, with at least one specific HPV-related diagnostic code as supplementary diagnosis.

Patient group III only consisted of patients from NPR, as this was the only data set which provided us with supplementary diagnosis (Appendix Figure 1). Patients in Patient group III were not included in the cost analysis.

**Additional file 1: Table S2** Diagnosis assignment methods, according to decision rules and corresponding tie rules and dataset application

| **Name** | **Decision rule** | **Tie rule** | **Applicable to dataset** |
| --- | --- | --- | --- |
| Diagnosis assignment A | For each patient, assign the most frequent main diagnosis across all years. | In the event of a tie in Diagnosis assignment A-D, the patient's last occurring diagnosis was assigned as the patient's main diagnosis. | NPR, KUHR, NorPD |
|  |  |  |  |
| Diagnosis assignment B | For each patient, assign the most frequent main diagnosis during the last calendar year. |  | NPR, KUHR |
|  |  |  |  |
| Diagnosis assignment C | For each patient, assign the most frequent main and supplementary diagnosis across all years. |  | NPR |
|  |  |  |  |
| Diagnosis assignment D | For each patient, assign the most frequent main and supplementary diagnosis during the last calendar year. |  | NPR |
|  |  |  |  |
| Diagnosis assignment E | For each patient, assign the most frequent main diagnosis during the first calendar year. | In the event of a tie in Diagnosis assignment E diagnosis, the patient's last occurring diagnosis the first calendar year was assigned as the patient's main diagnosis. | NPR, KUHR |
|  |  |  |  |
| Diagnosis assignment F | For each patient, assign the most frequent diagnosis over Diagnosis assignment A-E as the final main diagnosis. | In the event of a tie in Diagnosis assignment F, Diagnosis Assignment D was assigned as the patient's main diagnosis, Where Diagnosis assignment D did not apply, Diagnosis assignment B was assigned as the patient's main diagnosis. | NPR, KUHR |

Abbreviations: Abbreviations: KUHR, The Norwegian Control and Distribution of Health Reimbursement Database; NorPD, The Norwegian Prescription Registry; NPR, The Norwegian Patient Registry.

**Additional file 1: Figure S1** Assigning one diagnosis per patient in NPR, applying diagnosis assignment A-F.


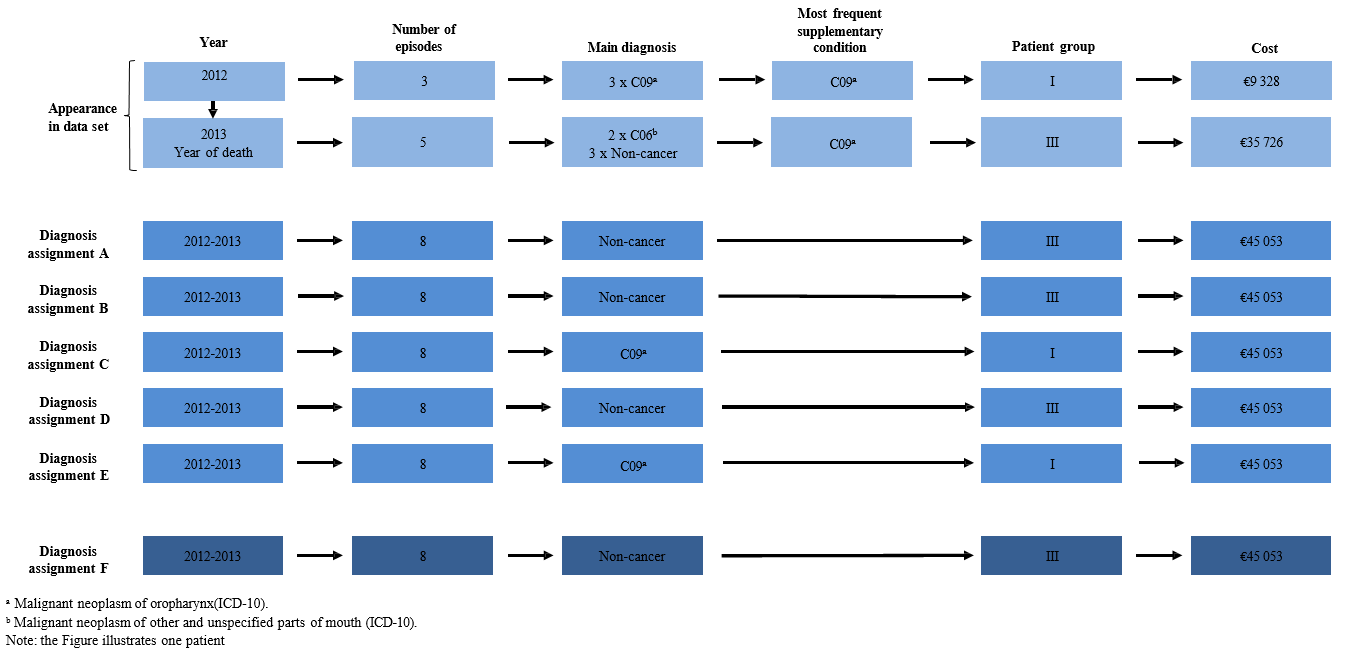


**Part III: Cost components**

**NPR costs**

The NPR data set included DRG-based costs, which does not account for costs related to laboratory- and radiology services, capital cost, administrative cost, pensions and research- and development costs. Unfortunately, the actual fixed costs associated to HPV-related cancers are unknown. Therefore, we added 22.17% of the DRG-based episode cost as a proxy for the omitted fixed costs (Appendix Table 3).

The rationale was based on estimates from Oslo Economics [4]:

- Cancer related diagnoses accounted for approximately €1 553 547 924 within the specialist care in 2014. The cost of laboratory and radiology services and other fixed cost was approximately €95 967 452 and €245 662 respectively. The cost of private specialists and DRG-based cost was approximately €12 564 317 and €1 199 353 835.
- When we excluded the cost associated with private specialists (as they are accounted for in KUHR in our analysis), we estimated that the cost of laboratory and radiology services and other fixed cost accounted for approximately 22.17% of the total cancer related cost within the specialist care in 2014.

**Additional file 1: Table S3** Description of cost components in NPR.

| Cost component | Description | Specification |
| --- | --- | --- |
| DRG unit cost | The average cost of average episode of care within the Norwegian specialist healthcare. Represent the 2014 DRG unit cost [5]. | €4 879 |
| Copayment | Patients' expenses by consumption of health services. Represents the 2014 patient copayment rate. The copayment is only triggered by outpatient episodes of care, not for day care and inpatient episodes of care [6]. | €38 |
| Cost per episode of care | The cost per single episode of care provided within the Norwegian specialist health care. The cost per episode of care is derived by multiplying the DRG unit price with the episode's respective DRG weight. Copayment is added when an outpatient episode of care is provided. | (DRG unit cost x DRG weight)  + copayment |
| Other costs | Costs not covered by the DRG unit cost, such as capital costs, pensions, research and development, outpatient laboratory and radiology services, and other non-activity based reimbursed services. The calculated percentage represents the share of the total somatic hospital costs which are not accounted for by the DRG costs. The is based on Oslo Economics’ [4] approximation. | 22.17% of the cost of the episode |
| Total cost per episode | Cost per episode when accounting for non-DRG based costs. Copayment is added for outpatient episodes of care. | [(DRG unit cost x DRG weight)/ 0.7793]  + copayment |

**KUHR costs**

The KUHR data set only included patient copayments and reimbursement costs. Primary care physicians and private specialist have additional fixed costs, which are not accounted for by the data set. To account for these, we added a cost of €16 to each episode of care provided by a primary care physician and €49 to each episode of care provided by a private specialist (Appendix Table 4).

Primary care physicians fixed cost per episode of care reflects the average per capita per episode of care. All primary care physicians receive a fixed annual cost per patient they are obligated to care for. In 2014, the per capita rate equaled €50 [7]. At the same time, the estimated total population in Norway per 2014 was

approximately 5 109 000 individuals [8]while regular consultations, simple contacts and home visits accounted for 16 060 192 of the total primary care physician consultations in 2014 [9]. Our estimated average per capita per episode of care reflects the total 2014 population multiplied with the 2014 per capita rate, dived by the total number of primary care physician consultations of interest in 2014.

Private specialists’ fixed cost per episode of care reflects the average practice allowance per episode of care. All private specialists receive an annual practice allowance to cover cost associated with buildings, medical devices and personnel. In 2014, the maximum practice allowance was €146 880. Size of the practice allowance depends on which class the specialists belong to [7]. We had no information about which class the specialists belonged to, and therefore assumed the specialists provided quite high resource consuming care as surgery, and applied the highest practice allowance. We further assumed that specialists on average had approximately 3 000 consultations per year2. The average practice allowance per episode of care was then the maximum practice allowance of 2014 divided by the average number of consultations per specialist.

**Additional file 1: Table S4** Description of cost components used in KUHR

| Cost component | Description | Specification |
| --- | --- | --- |
| Reimbursement fee  (Primary care physicians/private specialists) | Cost per contact reimbursed by the government to the provider of the service. The reimbursed amount depends on the type of contact. | - |
| Copayment | Cost per contact paid by the receiver of the service to the provider of the service. The copayment rate depends on the type of contact. | - |
| Per capita allowance per patient (Primary care physicians) | Allowance per patient funded by the government to the primary care physicians [7]. | €50 |
| Per capita allowance per episode of care (Primary care physicians) | The average per capita per episode of care. Per capita allowance multiplied with the total population divided by the number of consultations of interest. | €16 |
| Practice allowance (Specialist) | Allowance funded by the government to the specialist to cover expenses of buildings, personnel and medical devices. The cost reflects the maximum practice allowance[7]. | €146 880 |
| Practice allowance per episode of care (Specialist) | Average practice allowance per episode of care. Maximum practice allowance divided by the average number of consultations per specialist [10]. | €49 |

**NorPD costs**

Outpatient prescription costs include VAT, which is a transfer cost. Transfer costs are a societal cost, which we did not cover within the scope of analysis. To account for this, we divided the pharmacy retail price (PRP) by 1.25 (Appendix Table 5).

**Additional file 1: Table S5** Cost components used in NorPD

| Cost component | Description | Specification |
| --- | --- | --- |
| Pharmacy retail price | The total pharmacy retail price (PRP) per filled prescription at the dispensing time. | PRP per package x number of packages |
| Pharmacy retail price excl. VAT | In Norway all pharmaceutical have 25% VAT when purchased. VAT is a transfer and not a transaction, PRP is therefore excluded for the purpose of this analysis. | PRP/1.25 |

**Part IV: References**

1. WHO: **International classification of diseases and related health problems: Instruction manual. 10th revision.** . In*.* Edited by WHO. Geneva, Switzerland; 2016.

2. **ICPC-2. Den internasjonale klassifikasjonen for primærhelsetjenesten** [<https://ehelse.no/standarder-kodeverk-og-referansekatalog/helsefaglige-kodeverk/icpc-2-den-internasjonale-klassifikasjonen-for-primerhelsetjenesten>]

3. KITH: **Den internasjonale klassifikasjonen for primærhelsetjenesten**. In*.*, 1st edition edn. Trondheim, Oslo: Bjærum AS; 2004.

4. Oslo Economics: **Kreft i Norge: — kostnader for pasientene, helsetjenesten og samfunnet**. In*.* Oslo, Norway; 2016.

5. The Norwegian Directorate of Health: **Innsatsstyrt finansiering 2014**. In*.* Oslo, Norway: The Norwegian Directorate of Health,; 2013.

6. Forskrift om dekning av utgifter hos lege: **Forskrift om stønad til dekning av utgifter til undersøkelse og behandling hos lege**. In*.*; 2016.

7. The Norwegian Medical Association: **Normaltariff for avtalespesialister 2014-2015**

In*.* Norway: The Norwegian Medical Association; 2014.

8. Statistics Norway: **Dette er Norge 2014. Hva tallene forteller**. In*.* Oslo/Kongsvinger, Norway; 2014.

9. The Norwegian Directorate of Health: **SAMDATA kommune**. In*.* Oslo, Norway: The Norwegian Directorate of Health,; 2017.

10. The Norwegian Directorate of Health: **Aktivitetsdata for avtalespesialister 2014**. In*.* Oslo, Norway: The Norwegian Directorate of Health,; 2014: 9-10.
